# Effect of PR status on the prognosis of advanced ER-high HER2-negative breast cancer patients receiving CDK4/6 inhibitor combined with endocrine as first-line therapy

**DOI:** 10.1186/s12885-024-12621-y

**Published:** 2024-07-17

**Authors:** Lin Jia, Junning Peng, Nan Sun, Hongying Chen, Zhenyu Liu, Wenhui Zhao, Qingyuan Zhang, Liru Li

**Affiliations:** Department of Medical Oncology, Harbin Medical University Cancer Hospital, Harbin Medical University, Harbin, Heilongjiang 150081 China

**Keywords:** PR status, ER-high, Breast cancer, POD24, Prognosis

## Abstract

**Background:**

This study was designed to evaluate the effect of progesterone receptor (PR) status on the prognosis of advanced estrogen receptor (ER)-high human epidermal growth factor receptor 2 (HER2)-negative breast cancer patients receiving CDK4/6 inhibitor combined with endocrine as first-line therapy.

**Methods:**

Advanced ER-high HER2-negative breast cancer patients who were admitted to Harbin Medical University Cancer Hospital and received cyclin-dependent kinase (CDK)4/6 inhibitor combined with endocrine as first-line therapy were included for analysis. Patients were divided into PR-high group (11-100%), PR-low group (1-10%), and PR-negative group (< 1%) according to the expression of PR. Chi-square test was used to analyze the correlation of variables between groups. COX regression analysis were used to analyze the risk factors of survival. Kaplan-Meier survival curve was used to analyze the differences of progression-free survival (PFS) and overall survival (OS) between groups.

**Results:**

Among the 152 patients, 72 were PR-high, 32 were PR-low, and 48 were PR-negative. Compared with PR-negative group, the proportions of disease-free survival (DFS) ≥ 5 years and Ki-67 index ≤ 30% in PR-low group and PR-high group were significant higher. PR-negative patients were more likely to occur first-line progression of disease within 24 months (POD24) than PR-high(*P* = 0.026). Univariate and multivariate analysis showed that PR-negative and first-line POD24 occurrence were risk factors for survival. Survival curve analysis showed that compared with PR-high group, the PFS and OS were significantly lower in PR-negative group (*P* = 0.001, *P* = 0.036, respectively). Patients with first-line POD24 had shorter OS in the overall population as well as in subgroups stratified by PR status.

**Conclusions:**

PR-negative and first-line POD24 occurrence were risk factors of advanced ER-high HER2-negative breast cancer patients receiving CDK4/6 inhibitor combined with endocrine as first-line therapy. PR-negative patients had shortest PFS and OS. Regardless of PR status, first-line POD24 occurrence predicted shorter OS.

**Supplementary Information:**

The online version contains supplementary material available at 10.1186/s12885-024-12621-y.

## Introduction

Breast cancer is currently the most common cancer in women worldwide [1, 2], and its incidence is also increasing year by year. Hormone receptor(HR)-positive HER2-negative is the most common molecular subtype, accounting for about 60–70% of female breast cancer [3]. At present, according to the National Comprehensive Cancer Network (NCCN) guideline, CDK4/6 inhibitors combined with endocrine are recommended as first-line therapy for patients with ER-positive and HER2-negative advanced or metastatic breast cancer(MBC) [3]. However, studies have shown that patients with low expression of ER have similar biological characteristics and clinical outcomes to ER-negative patients, and have limited benefits from endocrine therapy [4], therefore, the choice of endocrine therapy for ER-low patients should be cautious. Based on a large number of clinical trial data, patients with ER-high and HER2-negative advanced breast cancer have clearly benefited from CDK4/6 inhibitor combined with endocrine therapy [5–9], but there are still some patients with poor prognosis. There is limited understanding of predictive markers for prognosis in combination with CDK4/6 therapy other than ER expression levels. Finding simple and reliable markers to predict the prognosis of CDK4/6 inhibitor combined with endocrine therapy is a critical clinical issue that needs to be solved immediately.

PR belongs to the steroidal hormone receptor family, which is the target gene of ER up-regulation, and its expression is dependent on estrogen [10]. It can be divided into two subtypes: PR-A and PR-B. PR-B is a transcription activator of target genes, while PR-A is an inhibitory factor with transcriptional activity and has an inhibitory effect on PR-B [11]. As a basic predictive marker and important prognostic factor for endocrine therapy [12], PR has been well known in early breast cancer and previous first-line treatment of advanced breast cancer, but whether it can predict the survival benefit of patients with CDK4/6 inhibitors combined with endocrine therapy is still limited.

Therefore, we investigated whether PR status exerted effect on prognosis of advanced ER-high HER2-negative breast cancer patients receiving CDK4/6 inhibitor combined with endocrine as first-line therapy in order to find simple and effective markers to predict the efficacy of CDK4/6 inhibitor.

## Methods

### Patients selection

The complete clinicopathological data of 152 patients with advanced ER-high and HER2-negative breast cancer who received CDK4/6 inhibitors and endocrine as first-line therapy in Harbin Medical University Cancer Hospital from January 2017 to December 2019 were collected. Patients received at least two cycles of systemic therapy and could be evaluated for treatment effect. All patients had biopsy pathology of recurrent or metastatic lesions, with comprehensive follow-up data and no loss to follow-up. The study protocol was approved by the Institutional Ethics Committee of Harbin Medical University Cancer Hospital, and has been performed in accordance with the ethical standards laid down in the 1964 Helsinki Declaration and its later amendments. All patients provided written informed consent for data use.

### Data collections

The clinicopathological data of patients were collected, including age, menopause, adjuvant therapy, DFS, initial diagnosis or recurrence of breast cancer, metastatic site, number of metastases, pathology of metastases, first-line endocrine drugs, first-line PFS and OS. Immunohistochemical staining was used to analyze ER, PR, HER2 and Ki-67 index in metastatic lesions by pathologists who were fully dedicated to breast cancer pathology. According to the American Society of Clinical Oncology (ASCO) /College of American Pathologists (CAP) guidelines, breast cancer samples with 11–100% of tumor cell nuclei positive should be interpreted as ER-high, ER-low was with 1-9% of cells staining. A sample would be considered ER-negative if < 1% or 0% of tumor cell nuclei were immunoreactive. Similar principles apply to PR testing. Patients were separated into three groups based on IHC result of PR staining: PR-high, PR-low and PR-negative. The efficacy of the treatment was evaluated by computerized tomography (CT). According to Response Evaluation Criteria In Solid Tumors (RECIST) 1.1, treatment-associated curative effects were classified into complete remission (CR), partial remission (PR), stable disease (SD), and progressive disease (PD) subtypes.

### Follow-up

Through the medical record review system and telephone follow-up of Harbin Medical University Cancer Hospital, the disease recurrence, metastasis and survival status of patients up to October 2023 were collected. DFS was defined as the time from the diagnosis of breast cancer to disease recurrence or metastasis. PFS was defined as the time from the initiation of first-line therapy to disease progression or the last visit or death, whichever came first. OS was calculated from the initiation of first-line therapy to the date of death or last follow-up.

### Statistical analysis

IBM SPSS program version 26.0 was used for data analysis. Normality test was performed on the measurement data, and the measurement data conforming to the normal distribution were described by x ± S. Chi-square test was used to analyze the correlation of variables between groups. Univariate and multivariate COX regression were used to analyze the risk factors for PFS and OS of advanced ER-high HER2-negative breast cancer. Kaplan-Meier method was used for survival analysis, and log-rank test was used to compare the disease-free survival rate between the two groups. Hazard ratios (HRs) together with 95% confidence intervals (CI) were provided. *P* < 0.05 was considered statistically significant.

## Results

### General data

**Fig. 1:**
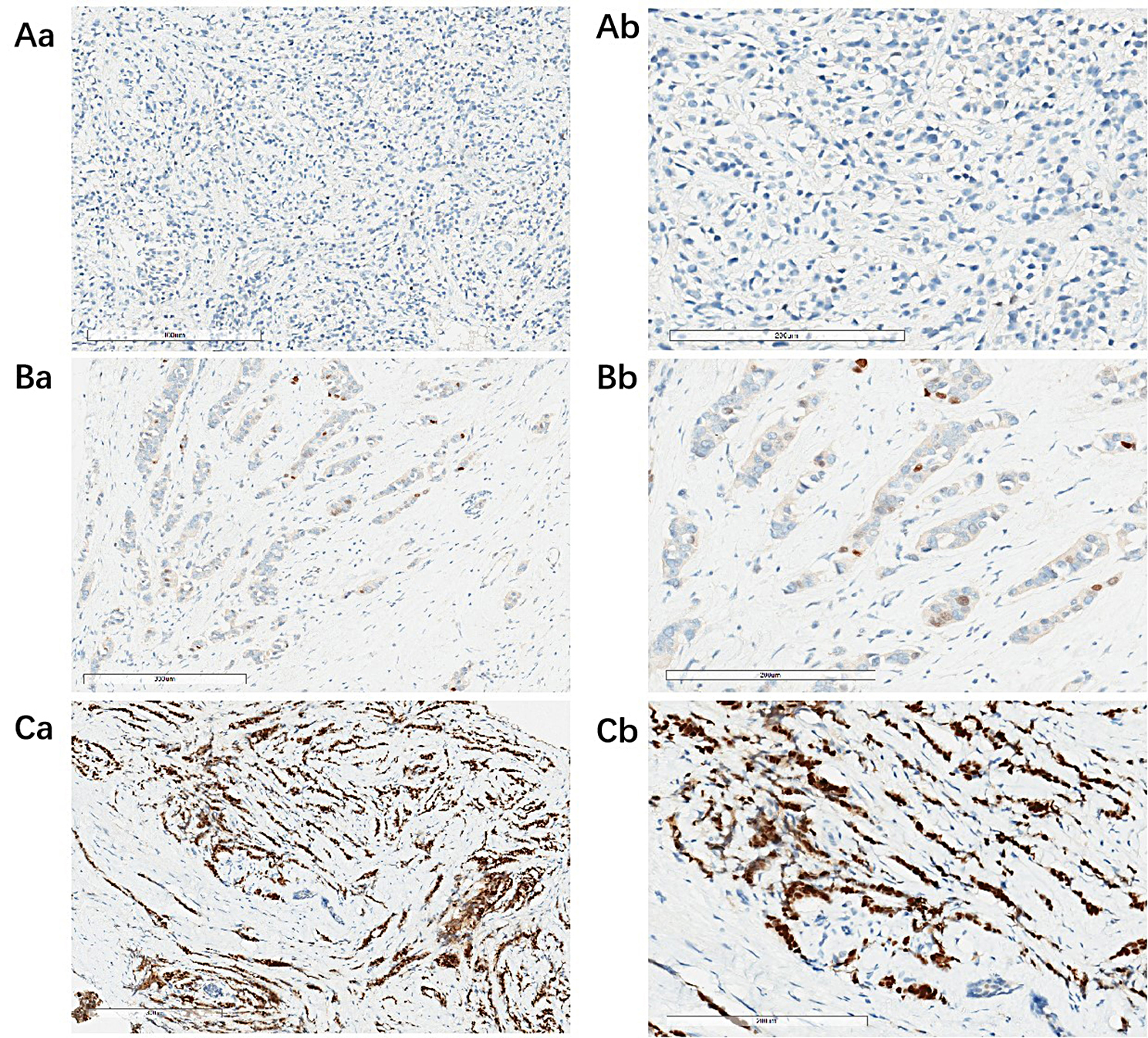
immunohistochemical staining of different PR expression. (**A**) Negative expression of PR (a:100×, b:200×).(**B**) 5%(low) expression of PR (a:100×, b:200×).(**C**) 90%(high) expression of PR (a:100×, b:200×)

The complete clinicopathological features of 152 patients with advanced ER-high HER2-negative breast cancer receiving CDK4/6 inhibitor combined with endocrine as first-line therapy were collected in this study. The age of patients ranged from 27 to 72 years, with a median age of 50.0 years. 96 (63.2%) patients were non-menopausal. 26 (17.1%) were treatment-naive stage IV. All patients had advanced biopsy pathology. Among the patients in the study, 72 (47.4%) were PR-high, 32 (21.1%) were PR-low, and 48 (31.6%) were PR-negative. Besides, 66 (43.4%) patients were with first-line POD24. Clinicopathological characteristics of the study participants are summarized in Table 1. The results of immunohistochemical staining of different PR expression are shown in Fig. 1.

**Table 1:** Clinicopathological characteristics of patients with advanced ER-high HER2-negative breast cancer

Characteristics	*N* = 152	%
**Median age (years)**	50.000 ± 11.280	
<60	116	76.3
≥ 60	36	23.7
**Menstrual status**		
Menopause	56	36.8
Non-menopause	96	63.2
**MBC status**		
Initial	26	17.1
Recurrent	126	82.9
**DFS(years)**		
<5	48	31.6
≥ 5	78	51.3
**Adjuvant endocrine therapy**		
Yes	78	51.3
No	48	31.6
**Adjuvant chemotherapy**		
Yes	102	67.1
No	24	15.8
**Adjuvant radiation therapy**		
Yes	34	22.4
No	92	60.5
**Number of metastatic sites**		
≤ 2	42	27.6
>2	110	72.4
**Metastatic sites**		
Visceral	90	59.2
Nonvisceral	62	40.8
**PR status**		
High	72	47.4
Low	32	21.1
Negative	48	31.6
**HER2 status**		
Zero	72	47.4
Low	80	52.6
**Ki−67 index**		
≤ 30%	116	76.3
>30%	36	23.7
**Endocrine therapy**		
Aromatase inhibitor	122	80.3
Fulvestrant	30	19.7
**First-line POD24**		
No	86	56.6
Yes	66	43.4
**OS (years)**		
>5	40	26.3
≤ 5	112	73.7

### Comparison of clinicopathological characteristics

The clinicopathological characteristics of the three groups were compared, and the results are shown in Table 2. Compared with PR-high and PR-low group, PR-negative group had a higher proportion of patients with DFS < 5 years (both *P* < 0.001), and no significant difference was observed between PR-high and PR-low patients (*P* = 0.597). Patients in PR-negative group had higher rate of Ki-67 index>30% (*P* = 0.018). 33.3% of PR-high patients, 43.8% of PR-low patients, and 58.3% of PR-negative patients were with first-line POD24 (PR-high versus PR-low *P* = 0.309, PR-high versus PR-negative *P* = 0.007, PR-low versus PR-negative *P* = 0.201). There were no significant differences in other clinicopathological features among the three groups.

**Table 2:** Clinicopathological characteristics of patients with different PR status

Characteristics	PR-high		PR-low		PR-negative		*P* value
	*N* = 72	%	*N* = 32	%	*N* = 48	%	
**Median age(years)**							0.068
<60	60	83.3	20	62.5	36	75.0	
≥ 60	12	16.7	12	37.5	12	25.0	
**Menstrual status**							0.984
Menopause	26	36.1	12	37.5	18	37.5	
Non-menopause	46	63.9	20	62.5	30	62.5	
**MBC status**							0.619
Initial	12	16.7	4	12.5	10	20.8	
Recurrent	60	83.3	28	87.5	38	79.2	
**DFS(years)**							<0.001 ^a^
<5	14	19.4	8	25.0	26	54.2	
≥ 5	46	63.9	20	62.5	12	25.0	
**Adjuvant endocrine therapy**							0.858
Yes	38	52.8	16	50.0	24	50.0	
No	22	30.6	12	37.5	14	29.2	
**Adjuvant chemotherapy**							0.185
Yes	44	61.1	26	81.3	32	66.7	
No	16	22.2	2	6.3	6	12.5	
**Adjuvant radiation therapy**							0.770
Yes	16	22.2	6	18.8	12	25.0	
No	44	61.1	22	68.8	26	54.2	
**Number of metastatic sites**							0.093
≤ 2	14	19.4	12	37.5	16	33.3	
>2	58	80.6	20	62.5	32	66.7	
**Metastatic sites**							0.445
Visceral	40	55.6	22	68.8	28	58.3	
Nonvisceral	32	44.4	10	31.3	20	41.7	
**Ki−67 index**							0.018 ^b^
≤ 30%	58	80.6	28	87.5	30	62.5	
>30%	14	19.4	4	12.5	18	37.5	
**HER2 status**							0.791
Zero	32	44.4	16	50.0	24	50.0	
Low	40	55.6	16	50.0	24	50.0	
**Endocrine therapy**							0.970
Aromatase inhibitor	58	80.6	26	81.3	38	79.2	
Fulvestrant	14	19.4	6	18.8	10	20.8	
**First-line POD24**							0.026 ^c^
No	48	66.7	18	56.3	20	41.7	
Yes	24	33.3	14	43.8	28	58.3	
**OS(years)**							0.108
>5	20	27.8	12	37.5	8	16.7	
≤ 5	52	72.2	20	62.5	40	83.3	

(a. PR-high versus PR-negative *P* < 0.001, PR-low versus PR-negative *P* < 0.001, PR-high versus PR-low *P* = 0.597. b. PR-high versus PR-low *P* = 0.388, PR-high versus PR-negative *P* = 0.028, PR-low versus PR-negative *P* = 0.014. c. PR-high versus PR-low *P* = 0.309, PR-high versus PR-negative *P* = 0.007, PR-low versus PR-negative *P* = 0.201.)

### Univariate and multivariate analyses

Univariate and multivariate analyses of clinicopathological characteristics affecting first-line PFS and OS of advanced ER-high HER2-negative breast cancer were performed by COX regression model. The variables included age, menstrual status, breast cancer status, DFS, adjuvant endocrine therapy, adjuvant chemotherapy, adjuvant radiotherapy, number of metastases, metastatic site, PR status, HER2 status, Ki-67 index, endocrine drugs, and first-line POD24 (only included in OS analysis).

Univariate analysis showed that DFS < 5 years, without adjuvant radiation therapy and PR-negative were risk factors of PFS in patients with advanced ER-high HER2-negative breast cancer (*P* = 0.027, *P* = 0.016, *P*<0.001, respectively. Table 3). Multivariate analysis showed that PR-negative was associated with shorter PFS (*P* = 0.013, Table 3). Univariate analysis showed that DFS < 5 years and PR-negative were associated with shorter OS in patients with advanced ER-high HER2-negative breast cancer (*P* = 0.039, *P* = 0.032, Table 4). Both univariate and multivariate analyses of risk factors for OS showed that first-line POD24 was associated with shorter OS (both *P* < 0.001, Table 4).

**Table 3:** Univariate and multivariate analysis of first-line PFS in advanced ER-high HER2-negative breast cancer

Variables	Univariate		Multivariate	
	HR(95% CI)	*P* value	HR(95% CI)	*P* value
**Age**	0.700(0.453–1.083)	0.109		
**Menstrual status**	0.886(0.591–1.328)	0.558		
**MBC status**	1.036(0.623–1.722)	0.892		
**DFS**	0.616(0.401–0.947)	0.027	0.783(0.491–1.250)	0.306
**Adjuvant endocrine therapy**	0.752(0.479–1.182)	0.217		
**Adjuvant chemotherapy**	0.819(0.487–1.378)	0.452		
**Adjuvant radiation therapy**	0.571(0.362–0.901)	0.016	0.621 (0.382–1.007)	0.053
**Number of metastatic sites**	1.183(0.780–1.795)	0.429		
**Metastatic sites**	0.813(0.553–1.196)	0.293		
**PR status**				
**low**	1.187(0.699–2.014)	0.526		
**positive**	2.213(1.431–3.421)	0.000	1.970(1.153–3.367)	0.013
**HER2 status**	0.764 (0.519–1.126)	0.174		
**Ki-67 index**	1.125(0.712–1.778)	0.613		
**Endocrine therapy**	0.695(0.439–1.099)	0.119		

(Annotation: Age<60 years, Menopause, Recurrent MBC, DFS ≥ 5 years, Without adjuvant endocrine therapy, With adjuvant chemotherapy, Without adjuvant radiation therapy, Number of metastatic sites ≤ 2, Visceral Metastasis, PR-negative, HER2 zero, Ki−67 index ≤ 30%, Endocrine therapy with aromatase inhibitor as references)

**Table 4:** Univariate and multivariate analysis of OS in advanced ER-high HER2-negative breast cancer

Variables	Univariate		Multivariate	
	HR(95% CI)	*P*	HR(95% CI)	*P*
**Age**	0.867(0.523–1.436)	0.578		
**Menstrual status**	0.866(0.548–1.368)	0.537		
**MBC status**	1.720(0.887–3.336)	0.108		
**DFS**	0.611(0.383–0.976)	0.039	0.882 (0.501–1.553)	0.663
**Adjuvant endocrine therapy**	0.952(0.587–1.543)	0.841		
**Adjuvant chemotherapy**	0.744(0.426–1.297)	0.297		
**Adjuvant radiation therapy**	0.729(0.441–1.205)	0.218		
**Number of metastatic sites**	0.605(0.358–1.023)	0.061		
**Metastatic sites**	1.292(0.824–2.026)	0.264		
**PR status**				
**low**	0.862(0.465–1.599)	0.637		
**positive**	1.684(1.045–2.714)	0.032	1.103(0.600−2.028)	0.753
**HER2 status**	0.839 (0.542–1.298)	0.430		
**Ki-67 index**	0.689(0.427–1.111)	0.126		
**Endocrine therapy**	0.846(0.500−1.434)	0.536		
**First-line POD24**	0.365(0.235–0.568)	0.000	0.340(0.206–0.564)	0.000

(Annotation: Age<60 years, Menopause, Recurrent MBC, DFS ≥ 5 years, Without adjuvant endocrine therapy, With adjuvant chemotherapy, Without adjuvant radiation therapy, Number of metastatic sites ≤ 2, Visceral Metastasis, PR-negative, HER2 zero, Ki−67 index ≤ 30%, Endocrine therapy with aromatase inhibitor, Without first-line POD24 as references)

### PFS and OS

**Fig. 2:**
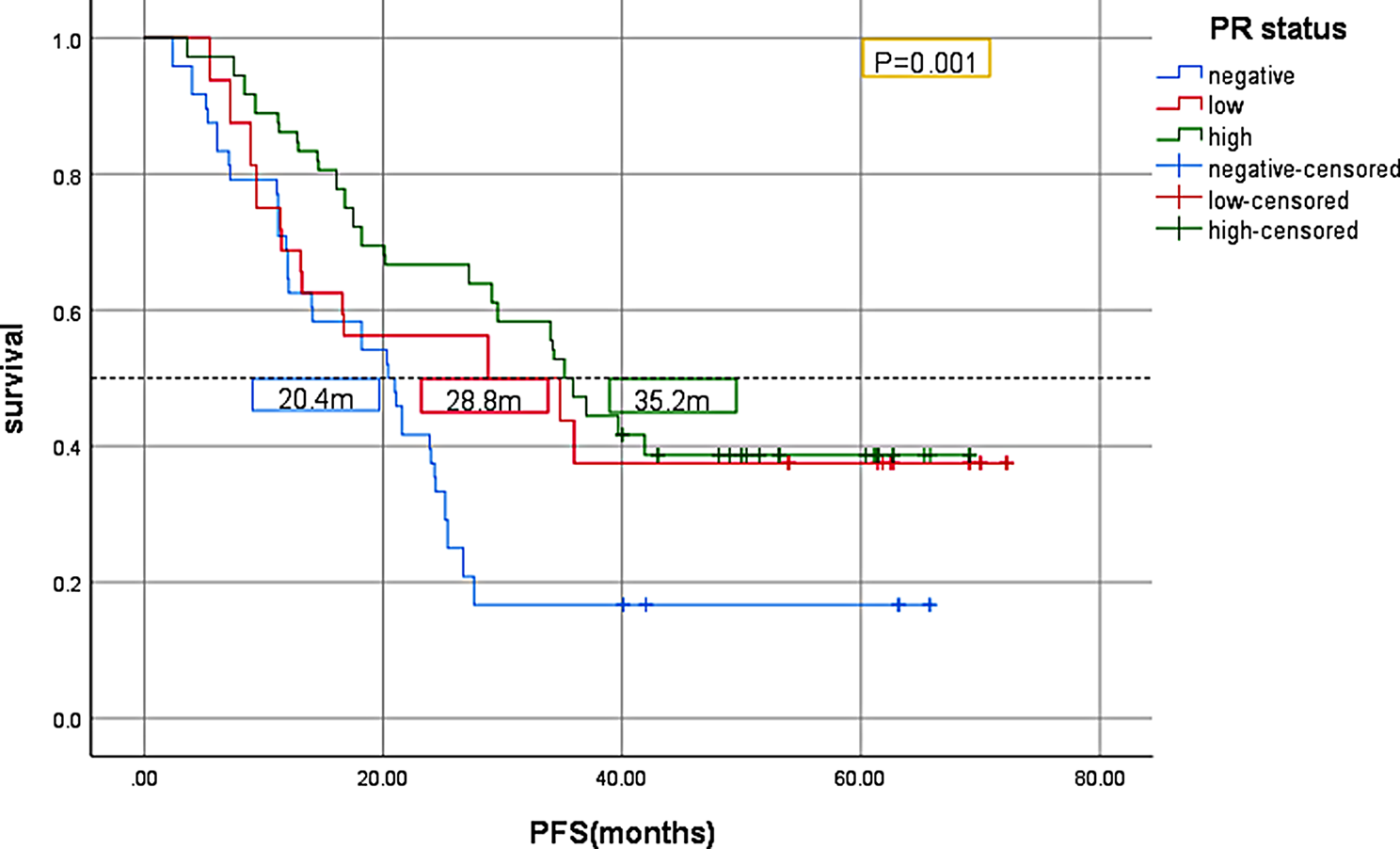
Kaplan-Meier PFS curves of advanced ER-high HER2-negative breast cancer

**Fig. 3:**
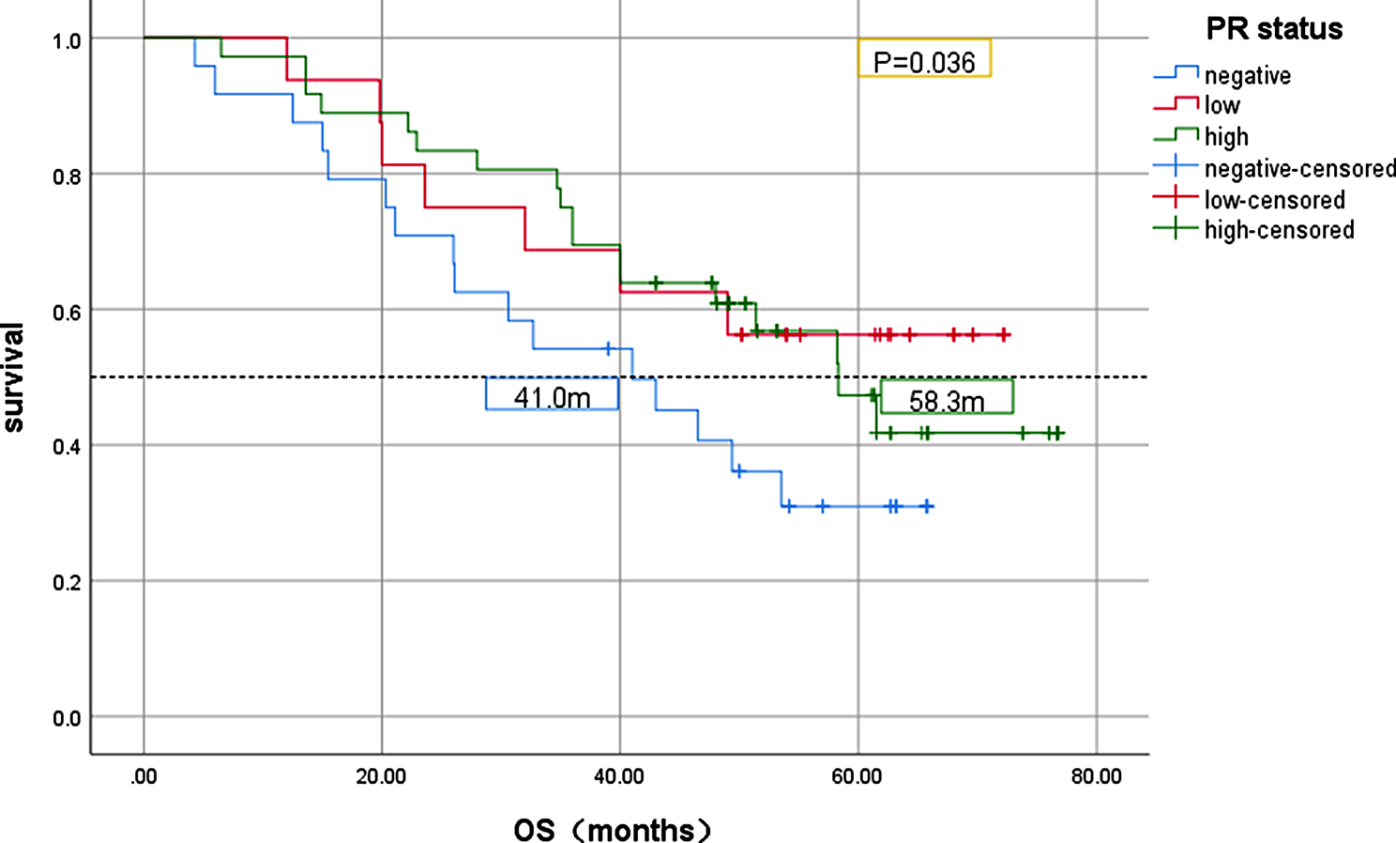
Kaplan-Meier OS curves of advanced ER-high HER2-negative breast cancer

**Fig. 4:**
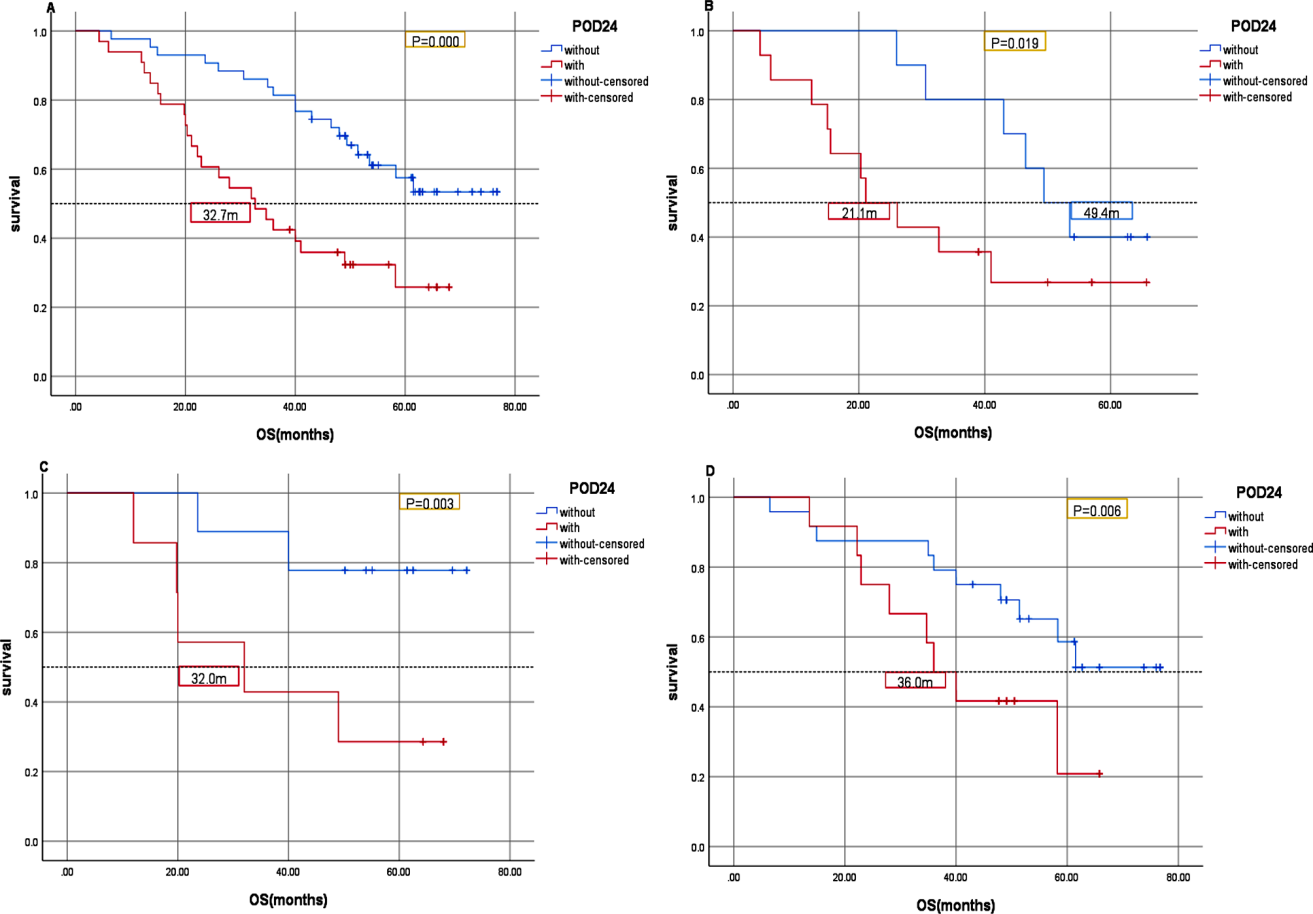
Kaplan-Meier OS curves of advanced ER-high HER2-negative breast cancer. (**A**). All patients. (**B**).PR-negative group. (**C**).PR-low group. (**D**). PR-high group

Kaplan-Meier analysis was used to analyze the relationship between PR status and survival in 152 patients with advanced ER-high HER2-negative breast cancer. The results showed that PFS in the PR-negative group was significantly lower than that in the PR-high group (*P* = 0.001, Fig. 2). Compared with the PR-high group, the median OS in the PR-negative group was significantly lower (*P* = 0.036, Fig. 3). In all patients enrolled, patients with first-line POD24 had shorter OS regardless of PR status(Fig. 4).

## Discussion

CDK4/6 inhibitors are a class of small molecule targeted drugs. CDK4 or CDK6 binds to cyclin D1 to form a cyclin D1-CDK4/6 complex, phosphorylates retinoblastoma protein (pRb) to release transcription factor E2F and drive cell cycle progression from G1 to S phase [13]. Blocking cyclin D1-CDK4/6-pRb signaling pathway can inhibit the proliferation of tumor cells [14]. CDK4/6 inhibitor combined with endocrine therapy is the standard first-line therapy for patients with HR-positive advanced breast cancer [3], and a large number of clinical trials have proved that it can improve the durability of response [5], prevent and overcome endocrine resistance of HR-positive breast cancer [6], and bring a better prognosis to patients [7–9]. However, the markers that can predict the prognosis after treatment are not fully clear [15, 16]. Due to the continuous in-depth study of ER status, it is generally believed that the characteristics of patients with low expression of ER are closer to triple negative [17], and the low expression of ER will affect the prognosis of patients treated with CDK4/6 inhibitors combined with endocrine therapy. In order to reduce the effect of ER status on prognosis, patients with ER-low expression were excluded in our study, and patients with ER-high expression were selected as study subjects.

As a basic predictive marker in endocrine therapy, the effect of PR expression on the prognosis after endocrine therapy has been studied in many cases, such as ER-positive, PR-negative breast cancer has a poor prognosis and is associated with endocrine resistance. Kurozumi et al. [18] pointed out that PR expression level is an independent prognostic factor for HR-positive and HER2-negative breast cancer patients, especially when Ki-67 expression level is between 10% and 30%, patients with PR < 20% have a worse prognosis. Piasecka et al. [19] found that PR-negative is a marker of increased EGFR activity, and the activation of EGFR can increase resistance to endocrine drugs. A European retrospective analysis of GEICAM/9906 study found that ER-positive /PR-negative breast cancer had stronger tumor proliferation, higher risk of recurrence and death, and worse survival outcomes [20]. In the study by Rocca A et al., in patients receiving only first-line endocrine therapy with an aromatase inhibitor, high PR (> 20%) was found to be independently associated with long time to progression in those with ER-high [21]. The prognostic value of PR status in CDK4/6 inhibitor combined with endocrine therapy is different. A pooled analysis by the FDA showed that all clinicopathological subgroups of patients with HR-positive, HER2-negative advanced breast cancer benefited from CDK4/6 inhibitors when combined with CDK4/6 inhibitors as first-line therapy, regardless of PR expression. However, the authors noted heterogeneity in the patients included in the analysis which may differ from the general population, the prognostic value of PR status on endocrine combined with CDK4/6 inhibitors still needs to be further studied [22]. In PALOMA-3, patients with PR-high expression showed longer benefits in both groups who received palbociclib plus fulvestrant or placebo plus fulvestrant [23]. Shao X et al. also found that PR ≥ 20% was associated with longer PFS in patients receiving a combination therapy with CDK4/6 inhibitors (8.5 vs. 6.7 months), and PFS was significantly shorter in the PR-negative/low cohort (*p* = 0.008) [24].

In this study, the clinicopathological characteristics of PR-high, PR-low, and PR-negative groups were compared. The results showed that compared with PR-negative group, the proportion of DFS>5 years and Ki-67 index ≤ 30% in PR-low and PR-high groups were significant higher (*P* < 0.001, *P* = 0.018). Compared with the PR-high group, the proportion of first-line POD24 occurrence in the PR-negative group was higher (*P* = 0.007), suggesting that PR-negative breast cancer patients were more likely to have disease recurrence within 5 years and disease progression within 2 years. COX regression analysis showed that DFS ≤ 5 years, without adjuvant radiation therapy and PR-negative were risk factors for PFS. First-line POD24 occurrence and PR-negative were risk factors for OS, indicating that the expression level of PR may affect the prognosis of patients. Further survival curve analysis also showed that the PFS and OS of the PR-negative group were significantly lower than those of the PR-high group (*P* = 0.001, *P* = 0.036), indicating that the prognosis of the PR-negative group was poor, which was similar to previous reports [24]. This study also found that the first-line PFS and OS of patients with PR-low expression were not statistically different from those of patients with PR-high expression, which may be related to the small number of patients with PR-low in this study. As we were able to collect data from only a single center and the number of patients using CDK4/6 inhibitors during the collection period was still limited by the high cost of the drug, this trial was still insufficient. Further studies with larger sample sizes are needed to analyze the prognosis difference between patients with PR-low and PR-high.

In addition, it was exciting to find that first-line POD24 occurrence was an independent risk factor for survival in the study population, independent of PR status. This analysis was mainly inspired by the study of the effect of POD24 on the prognosis of follicular lymphoma. Follicular lymphoma is a kind of inertia lymphoma, compared to other aggressive lymphoma, it is not easy to happen disease progression but hard to cure [25]. A large number of studies have proved when follicular lymphoma patients occur POD24, their prognosis are poorer, POD24 can be used as an independent predictor of prognosis of follicular lymphoma [26–28]. Compared with HER2-positive and triple-negative breast cancer, ER-positive HER2-negative breast cancer has a relatively ‘indolence’ of slow progression and good prognosis. Some patients with early-stage breast cancer can be cured, while those with advanced breast cancer are difficult to cure. Therefore, we analyzed the significance of first-line POD24 in advanced ER-high HER2-negative breast cancer patients, and our conclusion also verified that the prognosis of ER-positive HER2-negative breast cancer patients with first-line POD24 was worse and PR-negative patients were more likely to occur first-line POD24. This is the first time that the POD24 concept has been introduced into breast cancer, and its impact on breast cancer prognosis needs to be further investigated with a larger sample size. Meaningful, the effect of first-line POD24 on prognosis provides us with a new idea. We will continue to explore the effect of the time of first-line PFS on OS, and strive to provide more valuable prognostic indicators for patients.

In conclusion, PR-negative and first-line POD24 occurrence were risk factors of advanced ER-high HER2-negative breast cancer patients receiving CDK4/6 inhibitor combined with endocrine as first-line therapy. PR-negative patients had shortest PFS and OS. PR status may become a simple and accurate marker for predicting the efficacy of CDK4/6 inhibitors. For the first time, we found that the occurrence of first-line POD24 predicted shorter OS regardless of PR status. This is very important for clinical guidelines that doctors should use effective treatment as early as possible to improve the patient’s PFS and OS. However, this study still has some shortcomings, such as recall bias during patient follow-up and small sample size. With the increasing use of CDK4/6 inhibitors, more prospective studies are needed to confirm the effect of PR status and first-line POD24 on the prognosis of advanced ER-high HER2-negative breast cancer patients receiving CDK4/6 inhibitors combined with endocrine as first-line therapy.

### Electronic supplementary material

Below is the link to the electronic supplementary material.

Supplementary Material 1

## Data Availability

Data is provided within the manuscript and supplementary information files.

## Abbreviations

ASCO: American Society of Clinical Oncology
CAP: College of American Pathologists
CDK: Cyclin-dependent kinase
DFS: Disease-free survival
ER: Estrogen receptor
HER2: Human epidermal growth factor receptor 2
HR: Hormone receptor
HRs: Hazard ratios
MBC: Metastatic breast cancer
OS: Overall survival
PFS: Progression-free survival
POD24: Progression of disease within 24 months
PR: Progesterone receptor
pRb: Phosphorylates retinoblastoma protein
RECIST: Response Evaluation Criteria In Solid Tumors
